# Working Memory Capacity of Biological Motion’s Basic Unit: Decomposing Biological Motion From the Perspective of Systematic Anatomy

**DOI:** 10.3389/fpsyg.2022.830555

**Published:** 2022-03-22

**Authors:** Chaoxian Wang, Yue Zhou, Congchong Li, Wenqing Tian, Yang He, Peng Fang, Yijun Li, Huiling Yuan, Xiuxiu Li, Bin Li, Xuelin Luo, Yun Zhang, Xufeng Liu, Shengjun Wu

**Affiliations:** ^1^Department of Military Medical Psychology, Air Force Medical University, Xi’an, China; ^2^School of Computer Science and Engineering, Xi’an University of Technology, Xi’an, China; ^3^School of Information Technology, Northwest University, Xi’an, China; ^4^School of Martial Arts, Xi’an Physical Education University, Xi’an, China; ^5^School of Electronic and Information Engineering, Xi’an Jiaotong University, Xi’an, China

**Keywords:** biological motion, working memory capacity, systematic anatomy, change detection paradigm, Cowan’s formula, motion animation

## Abstract

Many studies have shown that about three biological motions (BMs) can be maintained in working memory. However, no study has yet analyzed the difficulties of experiment materials used, which partially affect the ecological validity of the experiment results. We use the perspective of system anatomy to decompose BM, and thoroughly explore the influencing factors of difficulties of BMs, including presentation duration, joints to execute motions, limbs to execute motions, type of articulation interference tasks, and number of joints and planes involved in the BM. We apply the change detection paradigm supplemented by the articulation interference task to measure the BM working memory capacity (WMC) of participants. Findings show the following: the shorter the presentation duration, the less participants remembered; the more their wrist moved, the less accurate their memory was; repeating verbs provided better results than did repeating numerals to suppress verbal encoding; the more complex the BM, the less participants remembered; and whether the action was executed by the handed limbs did not affect the WMC. These results indicate that there are many factors that can be used to adjust BM memory load. These factors can help sports psychology professionals to better evaluate the difficulty of BMs, and can also partially explain the differences in estimations of BM WMC in previous studies.

## Introduction

Biological motion (BM) refers to the holistic movement behavior of organisms in space (Johansson, 1973; Hoffman and Flinchbaugh, 1982; Kaiser et al., 2010; Jiang and Wang, 2011; Shen et al., 2014). Johansson (1973) was the first to utilize point-light displays (PLD), in which light bulbs were attached to 13 key joints on the body of a model in a black bodysuit. The motions of the model were photographed in a dark environment to obtain information about the movement of the joints. Since this method excluded visual information that were difficult to control at that time, it provided an effective method to acquire experiment materials for the study of BM, and has since been widely used in the study of BM perception (Chang and Troje, 2009; Kröger et al., 2014; Jaywant et al., 2016; Riddell et al., 2017), working memory of BMs (Shen et al., 2014; Ding et al., 2015; Gu et al., 2019; Liu et al., 2019; Guo et al., 2020), BM neurophysiology research (Grossman et al., 2000; Grossman and Blake, 2002; Kröger et al., 2014; Chang et al., 2021), and other fields. PLD enables rapid identification of human motion patterns (Johansson, 1973; Dittrich, 1993), gender (Mather and Murdoch, 1994; Troje, 2002; Troje et al., 2006), and person identification (Troje et al., 2005; Westhoff and Troje, 2007).

As social animals, human beings are driven to recognize and understand the behavior of other individuals from complex BMs every day. From infancy, motion information is very important when discriminating objects (Wood and Wood, 2021). The premise of understanding motion information is to store the BMs—the information carrier—in memory. Therefore, researchers have conducted a large number of studies on working memory. Smyth et al. (1988) and Smyth and Pendleton (1989) found that the working memory capacity (WMC) of movements was from 4 to 5 when there was no simultaneous interference task. Wood (2007) used computer-generated motion animations as experiment materials to measure the WMC of BM using the change detection paradigm, wherein participants were asked to repeat letters to suppress the verbal encoding of movement. Wood’s results showed that participants can maintain 2 to 3 BMs in their working memory. He then examined the visual WMC of the identities of agents and their motions, the result of BM WMC in this study were consistent with his previous study (Wood, 2008). Shen et al. (2014) used Wood’s experiment as a basis, but changed the experiment materials from computer-generated motion animations to PLD; changed the presentation method of the experiment materials from sequential to simultaneous; and changed the interference task from repeating letters to repeating numerals, which was deemed more suitable for Chinese participants. The results showed that the participants could remember at most 3.02 BMs (Shen et al., 2014).

Previous studies have basically proven that the WMC of BM is about 3; however, the following limitations can be noted in these studies: (1) The amount of experimental materials used has been small. Both Wood’s (2007) and Shen et al.’s (2014) studies utilized only seven experiment stimuli, with a maximum of five stimuli presented in a single trial, which may have caused the control of memory load to fail (as detailed by Gu et al., 2019). (2) BMs have been highly conceptualized. Most psychological studies of BMs have only used common actions in life as experiment materials (Vanrie and Verfaillie, 2004; Ma et al., 2005; Wood, 2007), and these motions can be summarized using very concise phrasing. Participants’ conversion of these motion patterns into verbal encoding could thus be done almost automatically, and simply repeating letters or numerals could not have completely inhibited participants’ verbal encoding. (3) The complexity of BM has been ambiguous. Working memory is a system that is used to store and manipulate limited information (Baddeley, 2012), and its capacity is affected by the complexity of that information. However, previous studies have ignored the influence of this factor on motion in WMC, which partially reduces the ecological validity of the studies’ results. (4) The BM information contained in the PLD has been incomplete (Kurz et al., 2020). BM is a typical non-rigid motion (Troje, 2013), and the power of movement comes from the contraction of skeletal muscles. When skeletal muscles contract, their shape and volume change—PLD cannot fully capture this information.

In addition to the above limitations, there is no unified scheme for classifying BMs in psychology, which forces former researchers to generally treat all BMs as homogeneous when studying BM working memory, ignoring the inherent characteristics of each BM. The WMC of these BMs can be better measured only if BMs are divided into several roughly related but relatively independent categories according to their inherent properties. Therefore, we hoped to find a common-sense and scientific way to classify BMs. As it happens, in anatomy (Bo and Ying, 2013; Standring, 2020), there is a set of internationally acknowledged, unified and standardized terms to describe human body shape and joint motions. By adopting these technical terms, we can comprehensively decompose each joint motion into its more fundamental components, which enables us to investigate the underlying features inherent in the BM. Thus, we suggest decomposing BM from the perspective of system anatomy. The concepts of axis and plane are artificially introduced, as follows: (1) The vertical axis is perpendicular to the ground, from top to bottom; (2) the sagittal axis is at an angle of 90° to the vertical axis, from the ventral side to the ventral side; and (3) the frontal axis is perpendicular to the above two axes, in the left and right directions, and parallel to the ground. Further, the three planes correspond to three axes, as follows: (1) the horizontal plane refers to the section that divides the human body into upper and lower parts; (2) the sagittal plane refers to the section that divides the human body into left and right parts, while the section that passes through the center of the human body is called the median sagittal plane; and (3) the frontal plane refers to the section that divides the human body into front and rear parts. The motion of joints is divided into translation, flexion and extension, adduction and abduction, rotation, and circulation. Translation comprises sliding between the two articular surfaces, such as the intercarpal joint. Flexion arises as the angle between the two bones of related joints decreases, while extension is an increase thereof. Medial rotation involves rotating the upper arm forward and toward the body, and lateral rotation entails rotating it back and away from the body; pronation consists of the forearm rotating the back of the hand forward, while supination comprises the back of the hand rotating backward. Circulation consists of the totality of flexion, adduction, extension, and abduction in sequence. Since circumflex movement can be further decomposed, it is not regarded as a basic unit of BM in this study. Using the above decomposition method, any BM can be decomposed into a combination of several basic units.

Here we planned to explore whether the BM WMC remain unchanged regardless of the difficulties inherent in BMs, and if not, what underlying anatomical factors or others might have effects through the following three experiments, and hoped to uncover some approaches to partially overcome the above limitations. Using computer-generated motion animations technique, a technology that has been used several times (Wood, 2007, 2008, 2010; Goldberg et al., 2015; Kenny et al., 2019; Kurz et al., 2020), by which we can strictly control concerned variables and remain irrelevant ones unchanged, thus enabled us develop more BMs with a low extend of conceptualization and then corroborate some hypothesis. First, we hypothesized that the difficulties inherent in the BMs bear some resemblance to each other but is not identical. The genesis of differences might lurk in joint, plate and duration involved in the BMs. In the view of above we conducted experiment 1, in which the concerned variables were strict controlled from the perspectives of systematic anatomy. Second, we hypothesized that BM WMC would not be affected by which side of the limb executes BMs. To confirm the above null hypothesis, we specifically designed an experiment 2 to perform an equivalence test, in which the BMs used were in one-to-one correspondence, the paired motions were identical in model, except for the limb executing the motion. Last but not least, we hypothesized that the more complex of the BMs the less the BM WMC. In the meanwhile, we also want to explore whether the effects of different types of articulation interference task on BM WMC remained unchanged. To this end, both variables above were controlled strictly in experiment 3, a 2 × 2 mixed designed experiment. BMs were divided into two groups in accordance with the anatomical complexity, and verbal articulation interference task was added to experiment 3 as a class of the interference task.

## Experiment 1: The Effect of Presentation Duration and Joint Use to Execute Motions on Working Memory Capacity of Biological Motion’s Basic Units

Experiment 1 was based on the experiment conducted by Shen et al. (2014). We decomposed BM into basic units to enhance the difficulty of verbal encoding within a limited time. By increasing the total number of experiment materials, it was more difficult for the participants to verbally encode the complete memory set, so as to avoid the failure of memory load.

### Methods

#### Participants

G*Power 3.1 software (Faul et al., 2009) was used to estimate the sample size. Under the premise of ensuring a medium effect size of 0.25, we set α = 0.05, 1–β = 0.80, and calculated the minimum sample size as 88. A total of 90 students from the Air Force Medical University in China were recruited to participate in the study. The student sample comprised 50 male and 40 female, with an average age of 20.12 ± 1.06 years. All participants were divided into six groups according to the between-group variables. They were all right-handed, or right preference mixed–handed (Li, 1983), with normal or corrected-to-normal vision, and naive to the experiment purpose. Informed consent was obtained prior to starting the experiment.

#### Experiment Design

This experiment adopted a 2 [duration of presentation (s): floating duration (2 × *n*), fixed duration (2)] × 3 (joint to execute motion: Elbow, shoulder, and wrist) × 5 (set size (*n*): 1∼5) mixed design, in which the presentation duration and joint used to execute the motion in question were between-group variables, and the set size was the within-group variable. BM working memory performance was the dependent variable.

We followed a variant of the change detection paradigm used by Shen et al. (2014), where the memory array stimuli were presented simultaneously (Fukuda et al., 2010). This variant can effectively overcome the influence of the serial position effect inherent in the sequent-presentation change detection paradigm on the participant’s working memory. The primacy and recency effects, widely reported in working memory related research (Agam and Sekuler, 2007; Wood, 2007; Berry et al., 2017), are major contributions to errors. In order to obtain a more accurate estimate, the simultaneous-presentation change detection paradigm was used to measure WMC in our three experiments.

#### Experiment Materials

In Experiment 1, we chose shoulder, elbow, and wrist movement as the observation object, and kept the hand joints at the distal end of the wrist from relative movement during the motion. Reciprocating motion animations were generated on different planes and different starting positions, respectively. A total of 30 motion animations were generated. All joint movements in Experiment 1 were executed using the right limb of the character model.

**FIGURE 1:**
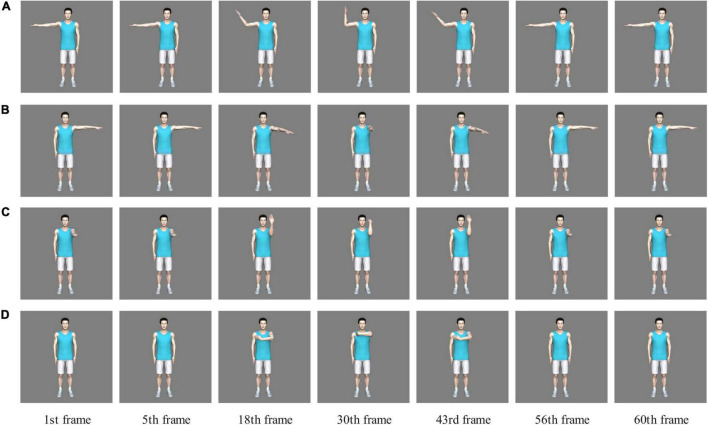
Part of the computer generalized motion animations. **(A)** A flexion-extension motion of the right upper limb’s elbow joint in the frontal plane. **(B)** An adduction-abduction motion of the left upper limb’s shoulder joint in the horizontal plane. **(C)** A flexion-extension motion of the left upper limb’s wrist and elbow joint in the sagittal plane. **(D)** A complex motion containing flexion-extension motion of the left upper limb’s wrist, elbow, and shoulder joint in the sagittal and horizontal plane.

Autodesk Maya 2015 three-dimensional modeling and animation software was used to create a human body model that could execute joint motion. The background of the final motion video was gray (RGB: 128, 128, 128), the animation duration was 2 s, the resolution was 240 × 240 pixels, and the frame rate was 30 frames per second. The first five frames kept the starting position unchanged; the 30th frame reached the intermediate stop position and the action was then reciprocated; the 56th frame returned to the starting position; and the last five frames kept the starting position still. Figure 1A shows the animation depicting the flexion-extension motion of the right upper limb’s elbow joint in the frontal plane; Figure 1B shows the adduction-abduction motion of the left upper limb’s shoulder joint in the horizontal plane; Figure 1C shows the flexion-extension motion of the left upper limb’s wrist and elbow joint in the sagittal plane; Figure 1D shows a complex motion containing flexion-extension motion of the left upper limb’s wrist, elbow and shoulder joint in the sagittal and horizontal plane. See Supplementary Videos 1–4 for motion animations corresponding to Figures 1A–D.

The BM experiment materials used in Experiment 1 were all executed by the right limb of the human model, including but not limited to Figure 1A. The BM experiment material shown in Figure 1B was used in Experiment 2, and the BM experiment materials shown in Figures 1C,D were used in Experiment 3, all of them did not appear in Experiment 1.

#### Experiment Instruments

The experiment stimuli were all presented on a 27-inch LED display with a resolution of 1,920 × 1,080 and a refresh rate of 60 Hz. The participants’ eyes were about 70 cm away from the screen, and the size of the motion animation on the screen was about 6.1° × 6.1°. In each trial, between one and five motion animations were randomly presented, distributed on an invisible circle with a radius of about 7.6° positioned at the center of the screen, and the background color was the same as motion video. All stimuli presentations and time controls were conducted using E-Prime 3.0 software.

#### Experiment Process

**FIGURE 2:**
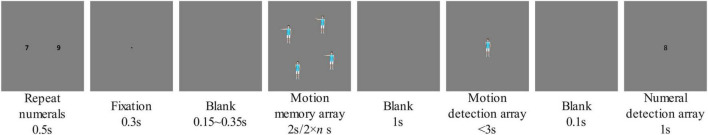
Experiment process. With set size = 4 shown as an example.

The experiment process is shown in Figure 2. First, two Arabic numerals were displayed on the screen for a duration of 500 ms. The distance between the two numerals was about 9.8°. The participants were asked to repeat these two numerals consistently and evenly during the trial until pressing a button in response to a numeral detection array. A 300 ms fixation point was then presented. After viewing an empty screen for 150–350 ms (this duration was random), between one and five motion animations were presented on the screen. Depending on the participant group, the duration of the animation was either 2,000 ms or (2,000 × *n*) ms (in the trial shown in Figure 2, if the participant was in the fixed-duration group the motion memory array was presented for 2,000 ms; if the participant was in the floating-duration group, the motion memory array was presented for 8,000 ms). After an empty screen was shown for 1,000 ms, a motion animation appeared in the center of the screen, and the animation stopped automatically after 2,000 ms, with the starting position kept unchanged for 1,000 ms. Participants were asked to judge whether the motion animation of the motion detection array was presented in the motion memory array via button response (“J” for present, “F” otherwise) given within 3,000 ms. If the participant did not make a button response within 3,000 ms, the numeral detection array would be automatically proceeded. The numeral detection array showed an Arabic numeral in the center of the screen. The participant was then asked to determine, within 1,000 ms, whether the number was one of the two numbers that had been repeated during the trial, again via button response (“J” for yes, “F” otherwise). Both the motion detection array and the numeral detection array had a 50% probability of showing a numeral or motion that had not appeared in the memory array.

The set size of the motion memory array contained a total of five levels. In the formal experiment there were 28 trials for each level, arranged in random order, every 20 trials consisted a group, and a 40-s rest between groups. Before taking part in the formal experiment, participants had to complete at least 10 practice trials to ensure their correct understanding of the experiment process. When the accuracy of the numeral detection array in the last 10 practice trials reached 0.8, the formal experiment was automatically entered. The entire experiment lasted for about 35 min.

#### Statistical Analysis

Excel 2019 and SPSS 26.0 were used for data collation and statistical analysis. When the accuracy of the participant’s numeral detection array exceeded the range of 2.5 times the standard deviation, they were eliminated from the analysis; a total of three participants were excluded based on this criterion.

The widely used Cowan formula in the change detection paradigm was utilized in this study (Wood, 2007; Shen et al., 2014; Guo et al., 2020). The formula is expressed as *k_*n*_ = n* × (*H*_*n*_—*F*_*n*_), where *n* stands for the set size, *k*_*n*_ for the WMC when the set size is *n*, *H*_*n*_ to the hit rate, and *F*_*n*_ to the false alarm rate. By incorporating the false alarm rate into the formula, we were able to correct for the influence of guesswork on the accuracy of results (Rouder et al., 2011), and more accurately estimate the individuals’ WMC.

Shen et al. (2014) showed that once the set size exceeds the individual’s WMC, the performance of working memory tasks under the change detection paradigm shows a downward trend. In order to more accurately estimate the individuals’ WMC of BM’s basic units, we experiment adopted *k*_*max*_ as the participants’ estimated WMC of BM.

### Results

#### Handedness Analysis of Participants

A chi-square test of the composition ratio of handedness was performed. Because more than 20% of the cells had a theoretical frequency of less than 5, Fisher’s exact test was performed. The results showed that there was no statistically significant difference in the composition of handedness among the various groups (Fisher = 13.466, *p* = 0.190).

#### Working Memory Capacity of Biological Motion of Each Group

**FIGURE 3:**
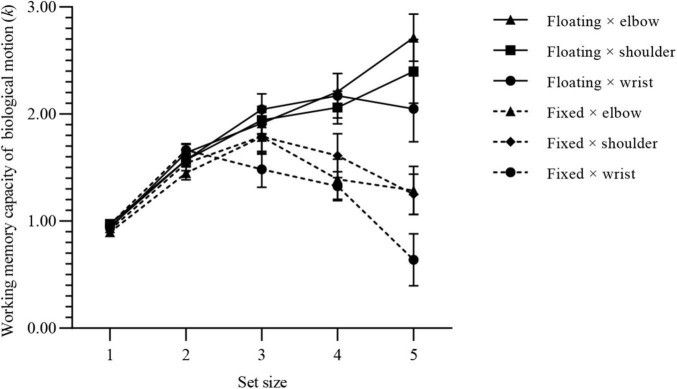
WMC of BM for each group of experiment 1. Values are reported as mean ± standard error.

After excluding the data for the three participants whose numeral detection array accuracy was lower than 2.5 times the standard deviations, 87 valid pieces of data remained, with an average of 22.87 (SD, 9.14) practice trials. For the floating-duration group, the accuracy of numeral detection arrays was 94.76% (SD, 0.42%); for the fixed-duration group, the accuracy of numeral detection arrays was 95.28% (SD, 0.40%). The WMC of BM of each group under the condition of different set sizes is shown in Figure 3.

Using *k*_*max*_ as a parameter to estimate the participants’ WMC of BM, we found that *k*_*max*_ (floating × elbow) = 2.95 (SD, 0.71), *k*_*max*_ (floating × shoulder) = 2.57 (SD, 0.59), *k*_*max*_ (floating × wrist) = 2.57 (SD, 0.63), *k*_*max*_ (fixed × elbow) = 2.06 (SD, 0.49), *k*_*max*_ (fixed × shoulder) = 2.08 (SD, 0.46), *k*_*max*_ (fixed × wrist) = 1.90 (SD, 0.45).

#### Mixed Analysis of Variance

The mixed analysis of variance (ANOVA) if the WMC of BM under the condition of different set sizes showed that *Mauchly W* = 0.15, *p* < 0.001, ε = 0.63 < 0.75. Therefore, the Greenhouse–Geisser method was used to correct the degrees of freedom. The main effect of set size was significant [*F*(2.51, 203.12) = 42.88, *p* < 0.001]; the main effect of presentation duration was also significant [*F*(1, 81) = 37.68, *p* < 0.001]; however, the main effect of joint used to execute motions was not significant [*F*(2, 81) = 1.22, *p* = 0.301]. There was a significant interaction between set size and duration of presentation [*F*(2.51, 203.12) = 24.14, *p* < 0.001], and a significant interaction between set size and joint used [*F*(5.02, 203.12) = 2.57, *p* = 0.028]; however, there was no significant interaction between the duration of presentation duration and joint used [*F*(2, 81) = 0.49, *p* = 0.616]. The interaction among the three was not significant [*F*(5.02, 203.12) = 0.72, *p* = 0.606]. Since the interactions between set size and duration of presentation, and between set size and joint used, were both significant, the simple effect was further investigated.

**FIGURE 4:**
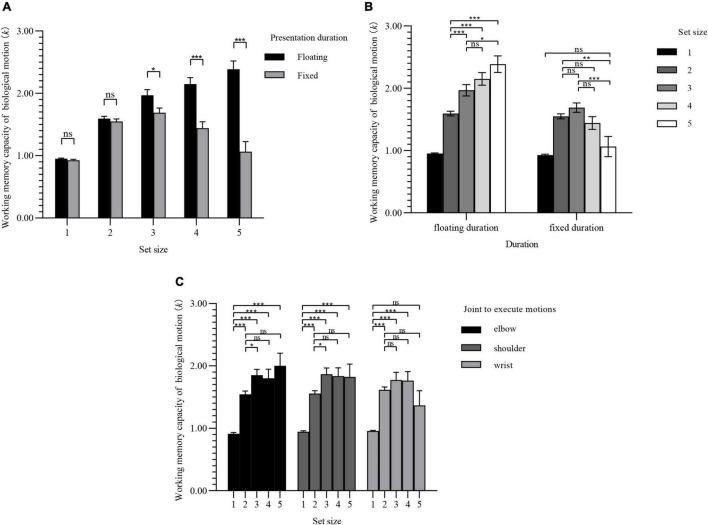
Simple effect results of experiment 1. Values are reported as mean ± standard error. The Bonferroni method was used for multiple test correction. **(A)** Comparison of BM WMC for presentation durations under different set sizes. **(B)** Comparison of BM WMC for set sizes under different presentation durations. **(C)** Comparison of BM WMC for set sizes under different joints to execute motions. ns: not significant; *p < 0.05; **p < 0.01; ***p < 0.001.

Figure 4A shows the comparison of the BM WMC for different presentation durations. When the set size was equal to 1 or 2, the difference in presentation duration had no effect on the WMC of BM. When the set size equaled 3 or above, the mean difference gradually increased, and the difference between groups was significant. When the set size was equal to 5, the presentation duration had the greatest impact on the WMC of BM. Under the condition of floating duration, the WMC of BM was 1.33 more on average than under the condition of fixed duration.

Figure 4B shows the comparison between the two presentation duration groups under different set sizes. When the set size equaled 1 or 2, there were significant differences compared to the other set sizes. When the set size reached 3 or above, the difference between groups disappeared. Under the condition of fixed duration, when the set size was lower than 3 the average difference mean value gradually increased and the differences were significant, and then began to decrease. When the set size arrived at 5, the difference between set size 1 group was no longer significant, and the WMCs of the two groups reached the same level.

Figure 4C shows the comparison of WMC BM when different joints were used to execute motions. The WMC of elbow and shoulder motions reached a higher level when the set size equaled 3, and then remained stable. The WMC of the wrist motions showed an inverted U-shaped change, which reached a peak when the set size was equal to 3, and then began to decrease.

### Discussion

In Experiment 1, we simultaneously presented a change detection paradigm supplemented by an interference task of repeating numerals to measure the WMC of BM’s basic units, and to explore the effect of presentation duration and joints used to execute motions thereon. The results showed that under the condition of floating duration, the WMC of BM’s basic units was significantly greater than that of the fixed-duration condition, which is consistent with the results of Shen et al. (2014). This indicates that, under the condition of fixed duration, the estimation of the WMC of BM was insufficient. There was no significant difference between the WMC of BM executed by different joints regardless of the presentation duration. Under the condition of floating duration, the WMC of BM was 2.5∼3.0; when it came to the fixed-duration condition, this decreased to 1.90∼2.10, and this result bear a close resemblance with Wood’s (2008) study.

## Experiment 2: The Effect of Handed or Non-Handed Limbs on the Working Memory Capacity of Biological Motion’s Basic Units

In addition to the joint factor that has been examined in experiment 1, limb is another significant anatomy factor that we are concerned about. In the meanwhile, a large number of previous studies have shown that there is a significant asymmetry in the performance of motor skills between the handed and non-handed limbs (Annett et al., 1974; Amunts et al., 2000; McGrath and Kantak, 2016). This asymmetry is also supported by findings from neuroimage studies of the motor cortex (Volkmann et al., 1998; De Lussanet et al., 2008; Michels et al., 2009; Suzuki et al., 2013). However, there are no studies that have examined whether BM WMC plays a part in the occurrence of this phenomenon. Based on the above two motivations, after examining the influence of joints on BM WMC in experiment 1, we further performed experiment 2 to examine the influence of limbs on BM WMC.

### Methods

#### Participants

The effectiveness analysis and sample size estimation of the two-sample mean equivalence test using a two-stage crossover design were performed by using PASS 15.0 software. A total sample size of 64 achieved 90% power at a 5.00% significance level when the true difference between the means was 0.00, the square root of the within mean square error was 0.51, and the equivalence limits were –0.30 and 0.30. All 45 participants in the floating-duration group in Experiment 1 were reenrolled in Experiment 2, and an additional 43 participants were recruited; of these 88 participants, five dropped out midway. Among the 83 participants who completed Experiment 2, 43 were male and 40 were female, with an average age of 20.52 ± 0.89 years. The experiment adopted a two-stage crossover design. Participants from Experiment 1 were included in the “right-then-left” group, and the rest were included in the “left-then-right” group. All participants were right-handed or right preference mixed–handed (Li, 1983), with normal or corrected-to-normal vision. Informed consent was obtained prior to starting the experiment.

#### Experiment Design

The experiment adopted a two-stage crossover design. After completing Experiment 1, participants in the right-then-left group accepted into the second stage at 2 weeks intervals, wherein the experiment materials comprised the left limb motion only. The left-then-right group followed the opposite process to that above. Cowan’s *k*_*n*_ was the dependent variable.

#### Experiment Materials

All 30 BM animations used in Experiment 1 were mirror-flipped to obtain the BM animations performed by the left limb. The duration, resolution, frame rate, and other parameters of the animation remained unchanged.

#### Experiment Instruments

The same instruments were used as in Experiment 1.

#### Experiment Process

The trial flow of Experiment 2 was the same as the trial process used in the Experiment 1 floating-duration group. In Experiment 2, the right-then-left group received the WMC test with all BMs executed by right limbs in the first stage, and the left-then-right group received the same test but with all BMs executed by left limbs. After a 2-week interval, the order was reversed in the second stage. Each stage lasted about 35 min.

#### Statistical Methods

Excel 2019, SPSS 26.0 and JASP 0.16 (JASP_Team, 2021) were used for data collation and statistical analysis. When the accuracy of the participant’s numeral detection array exceeded the range of 2.5 times the standard deviation they were eliminated from the analysis; a total of two participants whose data exceeded this standard were eliminated based on this criterion. Using the same Cowan formula as in Experiment 1, we calculated the WMC of BM’s basic units at different set sizes.

There is no similar previous study to refer to, we used the ratio method suggested by Laster and Johnson (2003) to determine the equivalence margin, α = 0.05, R_lower boundary_ = 100 (1–2α) %, R_higher boundary_ = 100 (1+2α) %, referring to the value of *k*_*max*_ under the condition of floating duration in Experiment 1, we convert the ratio to the effect value. Assuming that *k*_*max*_ as 3, the equivalence margin set by this method was 0.3.

In order to give how data quantitatively support a hypothesis (Wagenmakers et al., 2018a; Lakens et al., 2020), we also calculated the Bayes factor with JASP (Wagenmakers et al., 2018b).

### Results

#### Descriptive Analysis

After eliminating the data for two participants as detailed above, 81 valid cases remained. The average practice trial number in the first stage was 23.58 (SD, 10.40), and the accuracy of numeral detection arrays was 94.91% (SD, 2.66%). The average practice trial number in the second stage was 11.85 (SD, 4.22), and the accuracy of numeral detection arrays was 96.08% (SD, 2.23%). The differences between the number of practices (*t* = 9.40, *p* < 0.001) and the accuracy of numeral detection arrays (*t* = 3.02, *p* = 0.003) between the first and second stages were statistically significant. The Cowan’s *k*_*n*_ of the two experiment materials at different stages are shown in Table 1.

**TABLE 1:** Cowan’s *k*_max_ of the two experiment materials at different stages.

Stage	Experiment material	Mean	S.D.
1	Left limb	2.72	0.62
	Right limb	2.71	0.67
2	Left limb	2.77	0.64
	Right limb	2.86	0.82

#### Bayes Factor of Working Memory Capacity Between Handed and Non-handed Limbs

$H_{0}:\left|{\bar{k}}_{\max\cdot \mathrm{right}}-{\bar{k}}_{\max\cdot \mathrm{left}}\right|0.3$, $H_{1}:\left|{\bar{k}}_{\max\cdot \mathrm{right}}-{\bar{k}}_{\max\cdot \mathrm{left}}\right|0.3$. Because there is no prior knowledge, so we took Cauchy distribution with its scale parameter = 0.707 as the prior model. The equivalence paired *t*-test showed that there was no significance between−*k*_*max*⋅*right*_ and−*k*_*max*⋅*left*_ (*t* = 0.45, *p* = 0.653). And there was extremely strong evidence for *H*_0_ (BF_01_ = 338.22).

### Discussion

Experiment 2 investigated the difference in the WMC BM between the handed limb movement and the non-handed limb movement. Equivalence test results showed that when 0.3 was taken as the equivalent threshold, both one-side *t*-tests were rejected; thus, it can be considered that the WMCs of the BM’s basic units between groups were consistent. This indicates that whether the motions were executed by handed or non-handed limbs did not affect the WMC of the BM’s basic units.

## Experiment 3: Working Memory Capacity of Complex Biological Motions

Having examined the effects of joints and limbs on BM WMC, we planned to further examine whether the BM WMC would change under conditions that alter the anatomical complexity of the BM. Therefore, in Experiment 3, we divided BMs into two groups, according to the number of joints and planes contained in BM, in order to test the hypothesis that high-complexity BM is more difficult to remember.

Taking into account the fact that although we have strictly supervised the participants in Experiment 1 and 2 to complete the articulation interference task continuously and vocally, a number of participants still used verbal encoding strategies to remember our purposely designed low-conceptualized BMs, according to a simple survey after experiments.

Thus, in Experiment 3, we added repeating verbs as a new interference task, and added BM with multiple joints and motion planes as experiment materials. We conducted Experiment 3 to test the hypothesis that the WMC of complex BM was inferior compared to that of simple BM.

### Method

#### Participants

A total of 70 participants were recruited—53 male and 17 female, with an average age of 20.46 (SD, 1.46) years. According to the between-group variables, they were divided into four groups. All subjects were right-handed (Li, 1983), with normal or corrected-to-normal vision, and all participants were naive to the experiment purpose. Informed consent forms were collected.

#### Experiment Design

This experiment utilized a mixed design: 2 [complexity: A group (1 × 1| 1 × 2| 2 × 1), B group (2 × 3| 3 × 2| 3 × 3)] × 2 (interference task: repeat numerals, repeat verbs) × 6 [set size (*n*): 1–6], BM complexity and interference task comprised the between-group variance, set size was the within-group variance, and the dependent variable was the WMC BM *k_*n*_ = n* × (*H_*n*_—F_*n*_*).

#### Experiment Materials

A total of 60 BM animations were shown, of which 30 motions were executed by the right limb and the remaining 30 motions by the left limb. Motions executed by the respective limbs corresponded to and mirrored each other. According to the number of joints and motion planes involved in the BM, the BMs containing one joint one plane, one joint two planes, or two joints one plane were classified into group A; those containing two joints three planes, three joints two planes, or three joints three planes were classified into group B. Of the 108 candidate body action verbs, 23 with strokes or word frequencies exceeding 1.5 times the standard deviation were removed. This left 85 verbs, of which 15 with the highest word frequency were selected as repeating verbs to comprise the interference task materials. The final 15 verbs (listed in Table 2) had an average of 10.00 (SD, 1.96) strokes, and an average word frequency of 11.78/10^5^ (SD, 5.162/10^5^).

**TABLE 2:** Verbs repeated by the participant in Experiment 3.

Verbs displayed	Paraphrase	Verbs displayed	Paraphrase
摇	Shake	搬	Carry
抱	Embrace	插	Stick
挂	Hang	刮	Scratch
推	Push	握	Grasp
抽	Whip	捉	Grab
挤	Squeeze	搭	Build
挖	Dig	拣	Pickup
拖	Drag		

#### Experiment Instruments

The same instruments were used as in Experiment 1.

#### Experiment Process

The experiment process was the same as that used in the floating-duration group of Experiment 1. The set size contained six levels in total, and each level in the formal experiment comprised 24 trials, with a 40-s rest after every 24 trials.

#### Statistical Methods

Excel 2019 and SPSS 26.0 were used for data collation and statistical analysis. When the accuracy of the participant’s numeral/verb detection array exceeded the range of 2.5 times the standard deviation, they were eliminated from the analysis; a total of five participants were excluded based on this criterion. Under two interference task conditions, a mixed ANOVA was carried out on *k*_*n*_.

### Results

#### Descriptive Analysis

**FIGURE 5:**
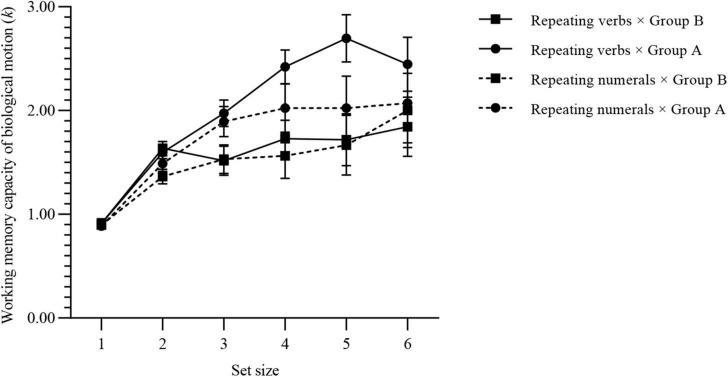
WMC of BM for each group of experiment 3. Values are reported as mean ± standard error.

The WMC of BM for each group is shown in Figure 5: *k*_*max*_ (verb × group A) = 3.27 (SD, 0.63), *k*_*max*_ (verb × group B) = 2.63 (SD, 0.83), *k*_*max*_ (numeral × group A) = 2.74 (SD, 0.82), *k*_*max*_ (numeral × group B) = 2.78 (SD, 0.98).

#### Mixed Analysis of Variance

Mauchly’s *W* = 0.06, *p* < 0.001, ε = 0.60 < 0.75. Therefore, the Greenhouse-Geisser method was used to correct the degrees of freedom. The main effect of set size was significant [*F*(3.02, 184.14) = 24.81, *p* < 0.001]; the main effect of complexity was significant [*F*(1, 61) = 8.68, *p* = 0.005]; the main effect of interference was not significant [*F*(1, 61) = 2.09, *p* = 0.153]; the interaction between set size and complexity was marginally significant [*F*(3.02, 184.14) = 2.37, *p* = 0.072]; the interaction between set size and interference was not significant [*F*(3.02, 184.14) = 0.60, *p* = 0.618]; the interaction between complexity and interference was not significant [*F*(1, 61) = 0.93, *p* = 0.339]; the interaction between these 3 variance was not significant [*F*(3.02, 184.14) = 0.73, *p* = 0.535]. Considering that this is an exploratory study, marginal significance can also make us more convinced of the alternative hypothesis to a certain extent (Pritschet et al., 2016), so we further conducted a mixed-design ANOVA under different interference task conditions.

#### Mixed ANOVA Under the Condition of Repeating Numerals

Under the condition of repeating numerals, Mauchly’s *W* = 0.04, *p* < 0.001, ε = 0.57 < 0.75. The same method was used to correct the degrees of freedom. The main effect of set size was significant [*F*(2.83, 79.34) = 8.47, *p* < 0.001]; but the main effect of complexity was not significant [*F*(1, 28) = 1.44, *p* < 0.240]; the interaction between set size and complexity was also not significant [*F*(2.83, 79.34) = 0.46, *p* = 0.700].

#### Mixed ANOVA Under the Condition of Repeating Verbs

Under the condition of repeating verbs, Mauchly’s *W* = 0.06, *p* < 0.001, ε = 0.58 < 0.75. The same method was used to correct the degrees of freedom. The main effect of set size was significant [*F*(2.92, 96.21) = 18.50, *p* < 0.001]; the main effect of complexity was significant [*F*(1, 33) = 10.705, *p* = 0.030]; and the interaction between set size and complexity was also significant [*F*(2.92, 96.21) = 3.13, *p* = 0.030]. The simple effect was further investigated.

**FIGURE 6:**
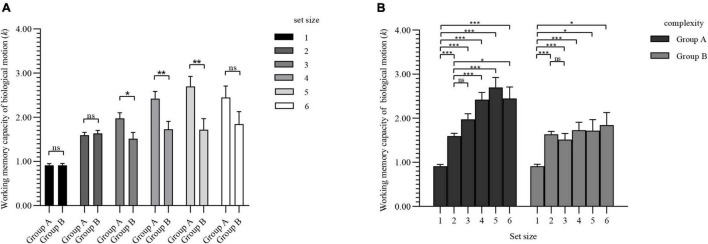
Simple effect results of experiment 3. Values are reported as mean ± standard error. The Bonferroni method was used for multiple test correction. **(A)** Comparison of BM WMC for two complexities under different set sizes. **(B)** Comparison of BM WMC for set sizes under different complexities. ns: not significant; *p < 0.05; **p < 0.01; ***p < 0.001.

Figure 6A shows the simple effect of complexities under the condition of different set sizes. Under the repeating verbs condition, when the set size ≥ 3, there was a significant difference in BM WMC between complexities.

The simple effect of set sizes under different complexities is shown in Figure 6B. The WMC of BM of group A increased as the set sizes increased, and there was a significant difference compared with the overall average increment. However, group B showed a steady trend after the set size reached 2.

### Discussion

Under repeating numerals conditions, complexity had no significant effect on WMC BM, but under repeating verbs conditions the negative effect of complexity on WMC BM was significant. This suggests that participants were affected by the repeating verb interference task when encoding group B’s BM. This indicates that the BM verbal encoding process consumed few cognitive resources; the simultaneously repeating numerals task could not completely suppress the verbal encoding of BM; and the repeating verbs interference task suppressed verbal encoding to a greater extent. Further cross-cultural research is needed to explore whether this phenomenon is limited to people that use ideograms like Chinese characters.

## General Discussion

From the perspective of systematic anatomy, this study conducted three experiments to identify the influencing factors of WMC of BM’s basic units.

This study adds the following to research related to BM working memory. First, we’ve explored the underlying anatomical factors which have effects on BM WMC. To our knowledge, this is the first time that anatomical basis has been used in psychology research to decompose BMs, thanks to the fact that this classification method is very mature, well-established and universally recognized in the field of anatomy (Bo and Ying, 2013; Standring, 2020), it makes it possible for us to apply it to the study of BM in the field of psychology, especially when it comes to motions executed by human body. Second, BMs used in our study was low-conceptualized and sufficient in amount due to the adoption of anatomical decomposing. The above two points make it more difficult for the participants in our experiments to reasonably encode BMs within a limit time, and partially overcome the imperceptible verbal encoding in the previous studies (Gu et al., 2019). It is a potential approach to further preclude the influence of the verbal encoding of the participants in the BM related studies in addition to the concurrent articulation interference task. Furthermore, the adoption of computer-generated motion animation enabled us to strictly control the irrelevant variables, including hairstyle, mutable expression and fluttering clothing, while a clear figure with non-rigid motion (Troje, 2013) can be completely preserved. This brought the behavior of the participants in our study closer to how they would react when they saw real actions, which can partially improve the external validity of the conclusions of our study.

### Effect of Presentation Duration on the Working Memory Capacity of Biological Motion’s Basic Units

The results of Experiment 1 indicate that under the fixed-duration condition, the WMC of BM’s basic units was underestimated; this is consistent with previous studies (Shen et al., 2014). Such findings might be attributed to the limited time given to participants, which caused them to fail to encode all BMs. The more BMs left uncoded, the more speculation may have occurred and the closer the resulting false alarm rate was to random levels. When the impact to WMC of false alarm rate was larger than that of set size, Cowan’s *k*_*n*_ began to drop. However, under the floating-duration condition, the decreasing tendency disappeared and the WMC was stable at a higher level. This shows that under floating-duration conditions, increases in the false alarm rate and the set size offset each other’s influence on Cowan’s *k*_*n*_.

### Effect of Joint Use on the Working Memory Capacity of Biological Motion’s Basic Units

Although there were no significant differences between the WMC of the BMs executed by the three joints, it is worth noting that the peaks of the WMC of the BM’s basic units did not appear at the same time. The BM executed by the wrist was the first to reach the peak of capacity, and then began to decline. The BMs executed by the elbow and shoulder peaked following the wrist joint, but Cowan’s *k*_*n*_ did not show a significant decrease. This indicates that the corrected recognition rate (Snodgrass and Corwin, 1988) for judging the BM executed by the wrist was lower than that of the elbow and shoulder when the number of BMs presented simultaneously was larger than three. As mentioned above, the trend of Cowan’s *k*_*n*_ reflected the change in false alarm rate under different conditions. It can thus be inferred that wrist movements are more difficult to remember, so individuals in our experiments had to guess when judging. As a result, when the set size exceeded the limit of the WMC of BM’s basic units, the degree of guessing with regard to the wrist motion was greater, which caused a rapid decrease in the WMC. This is in line with results found by Liu and Ku (2017). In short, the more BMs executed by wrist, the less accurate the memory. Our results also supported the discrete, fixed-resolution representation model of working memory (Zhang and Luck, 2008).

### Effects of Handed and Non-handed Limbs’ Motion on the Working Memory Capacity of Biological Motion’s Basic Units

The equivalence test results of Experiment 2 suggest that whether motion is executed by handed or non-handed limbs has no effect on the WMC of BM. A large number of previous studies have shown that there is significant asymmetry in the performance of motor skills in handed vs. non-handed limbs (Annett et al., 1974; Amunts et al., 2000; McGrath and Kantak, 2016). This asymmetry has also been found in neuroimage studies (Volkmann et al., 1998; Suzuki et al., 2013). Combined with the results of this study, the difference in the performance of motor skills learning between handed and non-handed limbs is mainly caused by physiological conditions, rather than differences in memory.

### Effect of Complexity and Interference Task on the Working Memory Capacity of Biological Motion

The results of Experiment 3 show that conducting the repeating verbs interference task simultaneously when measuring the complex BM WMC had a significant negative impact on the performance of the main task. This phenomenon was not found when participants memorized simple BMs, nor was it found under conditions of simultaneously repeating numerals. Previous studies have confirmed that simultaneous articulatory suppression tasks can inhibit individuals from verbal coding of BM (Vogel et al., 2001; Curby and Gauthier, 2007; Shen et al., 2014). The results of Experiment 3 suggest that the effect of the repeating verbs interference task in suppressing BM’s verbal encoding was inconsistent with the repeating numerals interference task. Repeating numerals could suppress the verbal encoding of BM in a relatively stable manner, regardless of the complexity of the BM to be maintained. However, the suppression effect of repeating verbs on verbal encoding of BM was regulated by the complexity of the BM.

The basic assumption of the dual-task paradigm is that if two tasks compete for the same limited cognitive resources, task performance will decline. Under the condition of repeating verbs, the WMC of simple BMs was significantly higher than that of complex BMs. This shows that complex BMs required more cognitive resources for verbal encoding, which means that complex BMs are more difficult to remember.

In these three experiments, we focused on the upper limbs with a greater range of motion (Bo and Ying, 2013; Standring, 2020), hoping to compromise between the representativeness and complexity of the experiment materials, and thus the trunk and lower limbs were irrelevant variables to control. This compromise results in limited external validity. BMs in previous studies required movements of various parts of the body to execute specific movement patterns or reach specific spatial locations. The BMs in our study only had upper limb movement, with the trunk and lower limbs remaining stationary, and had no specific purpose. Although some experiment results corroborated with previous studies (Wood, 2007, 2008; Shen et al., 2014), we are still unable to deduce whether the conclusion that BM WMC is associated with anatomical factors can be generalized to full-body motions. Therefore, we planned to investigate the effect of anatomy factors on the WMC of lower limb and trunk motions in future studies.

In summary, the results of this study suggest that: First, individuals can maintain two to three basic units of BM in working memory. Second, there is no difference between handed and non-handed limbs. Third, the more the wrist joint moves, the more inaccurate the memory of the upper-limb BMs. Finally, complex BMs are more difficult to remember. These results prompt us, BM WMC could be affected by inherent anatomical factors in BMs. In another word, it is unstable. Based on the conclusion of our research, we suggest that subsequent research on BM working memory should pay more attention to more detailed description and classification of the BMs used. In the meanwhile, this research can also provide a reference in the training of some certain populations, including gymnasts, diving athletes, martial arts athletes even pilots. In the process of training of these groups, coaches can modify the difficulty of movements they will demonstrate by controlling any of the above factors, through which the teaching progress may be mastered more objectively, and the training time for different movements can be set more targeted.

## Data Availability Statement

The raw data supporting the conclusions of this article will be made available by the authors, without undue reservation.

## Ethics Statement

The studies involving human participants were reviewed and approved by the Medical Ethics Committee of the First Affiliated Hospital of the Air Force Medical University. The patients/participants provided their written informed consent to participate in this study.

## Author Contributions

CW wrote the original draft. CW, YeZ, CL, and SW contributed to design of the study. WT translated and polished the first draft. YH, PF, and YL conducted part of the experiment. CW, HY, XiL, and BL performed the statistical analysis. XeL and YnZ gave valuable comments on the revision of experiment material. XfL and SW contributed to manuscript revision. All authors have read and agreed to the submitted version of the manuscript.

## Conflict of Interest

The authors declare that the research was conducted in the absence of any commercial or financial relationships that could be construed as a potential conflict of interest.

## Publisher’s Note

All claims expressed in this article are solely those of the authors and do not necessarily represent those of their affiliated organizations, or those of the publisher, the editors and the reviewers. Any product that may be evaluated in this article, or claim that may be made by its manufacturer, is not guaranteed or endorsed by the publisher.

## Abbreviations

BM: biological motion
WMC: working memory capacity.
